# Madness Or—What?

**Published:** 1894-08-04

**Authors:** 


### The Psychologist in History.

### MADNESS OR — WHAT?

### II.—The Terrible Czar.

Autocracy, whatever power it still may retain, is, practically speaking, on its last legs. Powerful as the Monarch of All the Russias may be, he would not dare, even did so pacific a prince desire, to exercise the authority, or to perpetrate the cruelties of a Czar of the past, Ivan the Terrible.

We do not speak of this Ivan as the ancestor of the present Emperor, for indeed the Romanoff dynasty, which he represents, did not come to the throne until, in the person of Ivan's son Feodor, the mythic dynasty of Rurik had come to an end. Ivan IV. came to the throne in 1553, when he was only three years old. It seems to have been his mother from whom he inherited the qualities that made his memory infamous. She was appointed Regent on her husband's death, but her cruelties and her irregular life brought about her murder at the end of four years. After that time one set of governors after another ruled and ravaged in the Emperor's name. His own friends and favourites were not spared, but by way of compensation he was allowed to torture animals to death, to trample women and children under his horse's hoofs, and to insult those who came before him as supplicants.

Yet the years that first ensued after he attained his majority were not unworthy. At that time Ivan was under three good influences—his wife Anastasia, a minister called Alexis Adashof, and a monk named Sylvester. Thirteen years these ruled him, and during this time Russia was prosperous. It was at this time that Archangel was founded, and trade with England begun. Printing was introduced, a new code of laws promulgated, and Turks and Tartars were kept in check. But these wars with national enemies, needful as they were, aroused his hereditary instincts. The merciful counsels of Adashof and Sylvester became distasteful to him; he was jealous of their intellectual power. In 1552 he met Vassian, a deposed bishop, who gave him more pleasing advice. "Keep no counsellor who is abler than yourself," said this subtle counsellor, "and you will reign unchecked. To a wiser man you will be a slave."

Opportunity soon came for practising the advice thus tendered. Anastasia died, and Sylvester and Adashof were accused of plotting her death. When they demanded to see the Czar and clear themselves they were refused admittance. One was imprisoned, the other banished to an ice-bound island in the White Sea, and Ivan was free to work his will. He was shrewd enough to use one section of his subjects in his designs against the rest. The object of his aversion was the boyars, or noblemen, who interfered with his authority. As they also tyrannised over those beneath them, the merchant classes were ready to support him. Thus a ruse he practised on the citizens of Moscow was entirely successful. In the winter of 1568 he withdrew to a village near Moscow, whence he sent a letter to be read by the Bishop of Moscow from the steps of the altar. He assured the citizens and merchants of his goodwill towards them, but complained of the wrongs that had been done in his minority, and announced his determination to renounce the empire. To the people of Moscow this seemed to be leaving them at the mercy of the boyars, and they besought him to return, and readily enlisted in his service. Thus he was enabled to raise a bodyguard of six thousand horsemen, who carried a dog's head and a brush at their saddle bows to indicate their determination to worry to death and sweep away the enemies of the Czar. These mercenaries ravaged the boyars' lands, reduced them to beggary, and drove their families out to perish in the snow. The Czar was satisfied.

After this the Emperor's cruelty became unbounded. Some courtesy shown to his cousin Vladimir so enraged the suspicious tyrant that he not only killed those who had offered it, but poisoned Vladimir himself, his wife Evdoxia, their four children, and all their attendants. Ivan was a connoisseur in the art of poisoning. He had prepared for him a poison which could be graduated to kill with different degrees of swiftness, and is said to have slain a hundred men in testing it. Inevitably he, like all such miscreants, lived in an atmosphere of terror, and any rumour of revolt was believed implicitly and punished brutally. Thus, when a criminal who had been punished for some slight offence by the municipality of Novgadd pretended to have discovered a conspiracy there, his word was accepted, the Czar and his mercenaries plundered the town, killed the horses and cattle, destroyed such booty as they could not carry away, and murdered, with every variety of torture a diseased imagination could invent, about 27,000 persons. Then in a spirit that would have been that of the bitterest satire in a saner man, but was wholly sincere in him, he ordered the survivors to pray for him and his Christian army.

One man alone, Philip, the Metropolitan of Moscow, dared to speak the truth to him about his cruelties. For this the bishop, in his seventy-ninth year, was deposed, insulted, imprisoned, and finally murdered. In the end Ivan murdered his own son in a fit of passion, and though, in his remorse, he sent large sums to all the patriachs of the Greek Church to be spent in prayers for his soul, he showed no greater regard for the lives of his subjects than before. Mutilations were his jests, tortures his amusements. To outrage the emotions of humanity was one of his most refined pleasures. He compelled a brother to kill a brother, a son to be the executioner of his father. His humour showed itself in making wives eat their meals opposite their husband's corpses, and mothers dwell for days in presence of their murdered children.

Such was Ivan the Terrible, a man by nature selfish and cruel, from whom absolute power removed the safeguards that protect ordinary humanity from itself, and developed into disease all his innate weaknesses. Yet when he died his people wept for him. "Their patience had no limits," says Karamsin, the Russian historian, as quoted in Dr. Ireland's "Blot on the Brain," "for they regarded the rule of the Tsar as the rule of God, and held all contradiction as a transgression of His law. They perished, but they saved for us the strength of Russia; for in the depth of the obedience of the people lies the strength of the empire."

Thus says the modern commentator, Is it wonderful that despotism has its last stronghold in the Russian empire?

